# Clinical evaluation of neuroinflammation in child-onset focal epilepsy: a translocator protein PET study

**DOI:** 10.1186/s12974-020-02055-1

**Published:** 2021-01-06

**Authors:** Kuriko Kagitani-Shimono, Hiroki Kato, Ryoko Kuwayama, Koji Tominaga, Shin Nabatame, Haruhiko Kishima, Jun Hatazawa, Masako Taniike

**Affiliations:** 1 Department of Child Development, United Graduate School of Child Development, Osaka University, Suita, Osaka Japan; 2 Department of Pediatrics, Osaka University Graduate School of Medicine, Suita, Japan; 3 Epilepsy Center, Osaka University Hospital, Suita, Japan; 4 Department of Nuclear Medicine and Tracer Kinetics, Osaka University Graduate School of Medicine, Suita, Japan; 5 Department of Neurosurgery, Osaka University Graduate School of Medicine, Suita, Japan; 6 Department of Quantum Cancer Therapy Research Center for Nuclear Physics, Osaka University, Suita, Japan

**Keywords:** Epilepsy, Neuroinflammation, Microglia, Positron emission tomography, TSPO

## Abstract

**Background:**

Neuroinflammation is associated with various chronic neurological diseases, including epilepsy; however, neuroimaging approaches for visualizing neuroinflammation have not been used in the clinical routine yet. In this study, we used the translocator protein positron emission tomography (PET) with [^11^C] DPA713 to investigate neuroinflammation in the epileptogenic zone in patients with child-onset focal epilepsy.

**Methods:**

Patients with intractable focal epilepsy were recruited at the Epilepsy Center of Osaka University; those who were taking any immunosuppressants or steroids were excluded. PET images were acquired for 60 min after intravenous administration of [^11^C] DPA713. The PET image of [^11^C] DPA713 was co-registered to individual’s magnetic resonance imaging (MRI), and the standardized uptake value ratio (SUVr) in regions of interest, which were created in non-lesions and lesions, was calculated using the cerebellum as a pseudo-reference region. In the case of epilepsy surgery, the correlation between SUVr in lesions and pathological findings was analyzed.

**Results:**

Twenty-seven patients (mean age: 11.3 ± 6.2 years, male/female: 17/10) were included in this study. Of these, 85.1% showed increased uptake of [^11^C] DPA713 in the focal epileptic lesion. Three patients showed epileptic spasms, suggesting partial seizure onset, and all 18 patients with abnormal lesions on MRI were similarly highlighted by significant uptake of [^11^C] DPA713. DPA713-positive patients had a broad range of etiologies, including focal cortical dysplasia, tumors, infarction, and hippocampal sclerosis. Five out of nine MRI-negative patients showed abnormal [^11^C] DPA713 uptake. The SUVr of [^11^C] DPA713 in lesions was significantly higher than that in non-lesions. In seven patients who underwent epilepsy surgery, increased [^11^C] DPA713 uptake was associated with microglial activation.

**Conclusions:**

This study indicates that [^11^C] DPA713 uptake has valuable sensitivity in the identification of epileptic foci in child-onset focal epilepsy, and inflammation is implicated in the pathophysiology in the epileptic foci caused by various etiologies. Further research is required to establish diagnostic tools for identifying focal epileptogenic zones.

**Supplementary Information:**

The online version contains supplementary material available at 10.1186/s12974-020-02055-1.

## Background

Epilepsy, a common chronic neurological disease, affects approximately 65 million people worldwide [1] and is the third contributor to the global burden in neurological disorders [2]. Etiologies, such as genetic structural abnormality, infection, metabolic abnormality, and autoimmune encephalitis [3], have been recognized, while the common pathophysiological mechanisms include (1) increased excitatory neurotransmission and/or reduced inhibitory neurotransmission at the epileptogenic zone and (2) recurrent seizures that may lead to further epileptogenesis or progression of epilepsy. Therefore, the first-line choice for epilepsy is antiepileptic drugs that modify the imbalance between excitatory and inhibitory neurotransmission or suppress ictogenesis. In the last two decades, many new antiepileptic drugs have been developed; however, approximately 30% of patients still have refractory prognosis [4].

Accumulating evidence indicates that the inflammatory process is a strong trigger and modulator in the epileptic brain. For instance, kainic acid-induced seizure mice (a model of temporal epilepsy) showed activated microglia shortly after acute seizures [5] [6], and human tissues from the resected foci of refractory epilepsy exhibited reactive microglia [7–9]. Rasmussen’s encephalitis and other encephalitis-associated epilepsies are well known as inflammation-associated epilepsies [10]. Accordingly, some anti-inflammatory agents, such as corticosteroids, immunoglobulins, and immunosuppressants, are the most effective therapies for epileptic encephalopathies [11]. Because neuroinflammation plays an important role in epilepsy and other neurodegenerative disorders, specific in vivo markers for neuroinflammation are needed for therapeutic purposes.

Positron emission tomography (PET) imaging using translocator protein (TSPO)-binding radioligands is the most widely used method to assess microglial activation in patients. TSPO, an 18-kDa translocator protein, which is located on outer mitochondrial membranes, has been demonstrated to be a peripheral benzodiazepine receptor [12] and has been consistently observed in activated microglia [13]. In the past two decades, because of a high requirement for the detection of neuroinflammation in vivo, numerous TSPO PET ligands have been developed, and they were classified into the first- and second-generation ligands. ^11^C PK11195 is the most widely used first-generation ligand but has some disadvantages, such as high non-specific binding and low brain bioavailability [14]. Several second-generation ligands, such as ^11^C-PBR28, ^11^C-DPA713, ^18^F-PBR111, and ^18^F-DPA714, have been developed to improve the signal to noise ratio, although low-affinity is still a problem in participants with rs6971 polymorphism (Ala147Thr) in the TSPO gene [15].

Neuroinflammatory pathophysiology has been investigated using those TSPO ligands in human neurological conditions. For instance, both the acute dysmyelinating lesion and secondary progressive white matter lesion in multiple sclerosis showed higher TSPO radioligand uptake [16–18], and ^11^C-DPA713 uptake was more sensitive to and showed better correlations with microglial activation at the acute and chronic phases after stroke [19]. Moreover, high TSPO distribution was associated with post-treatment Lyme disease syndrome [20] and clinical stage and extent of tau aggregation in patients with Alzheimer’s disease [21], and patients with traumatic brain injury, especially repeated injuries, showed higher TSPO radioligand uptakes in the atrophic brain regions compared to the controls [22]. These findings further support the usefulness of TSPO PET in the detection of neuroinflammation in neurological disorders.

Notably, many studies using TSPO PET were performed in adult patients, except for one study where age-related changes in TSPO distribution were examined in only ten children with normal TSPO distribution [23]. Therefore, it remains unclear whether there are differences in TSPO distribution between child and adult epilepsy, and few studies have investigated the usefulness of TSPO PET in child-onset epilepsies, including epileptic encephalopathies. Therefore, this study aimed to examine (1) the usefulness of TSPO PET in detecting the epileptogenic zone in child-onset epilepsy using [^11^C] DPA713; (2) the factors that influence TSPO distribution, such as age, etiology, and seizure frequency; and (3) the consistency between histological examinations of surgical specimens and TSPO uptake among various etiologies.

## Methods

### Participants

Patients with intractable focal epilepsy aged more than 1 year were recruited at the epilepsy center of Osaka University Hospital between December 2016 and March 2019. Patients who were taking any immunosuppressants or steroids or were at risk of becoming pregnant were excluded from the study. In all patients, except one, who had controlled seizure, seizure types were diagnosed using video-encephalography (video-EEG) (EEG1200; Nihon-koden). Furthermore, the epileptogenic focus was identified with seizure semiology, ictal video-EEG, and magnetoencephalogram (RICOH), and confirmed by structural magnetic resonance imaging (MRI) (3T Discovery MR 750w system, GE Healthcare), and ^18^F-2-deoxy-2-fluoro-d-glucose PET (FDG-PET) (Eminence SOPHIA SET-3000 GCT/X, Shimadzu Co). Seven patients did not undergo FDG-PET based on the attending physician’s judgment. All participants were checked for the TSPO binding polymorphism by genotyping of re6971 (A/A: high-affinity; A/T: mixed-affinity; and T/T: low-affinity). Seizure frequencies were classified as yearly (one to eleven times/year), monthly (one to four times/month), weekly (two to six times/week), and daily (more than one time/day).

### MRI data acquisition

We performed 3T-MRI(Discovery MR750W, GE Healthcare)with a 24-channel-head coil using the following parameters: silent T1W; TR/TE = 880/0.016 ms; FOV = 240 mm; Matrix = 240 × 240; slice thickness, 1.0 mm; gap, 0.5 mm; 280 slices; acquisition time, 5 m 10 s.

### PET imaging and analysis

[^11^C] DPA713 was prepared using the SUMITOMO Gas-Phase Synthesizer C-GPS 100 system at the Radiopharmaceutical Laboratory of Osaka University Hospital. Mean molar activity at the end of synthesis was 141.0 ± 36.6 GBq/mmol (range 53.5–192.1 GBq/mmol).

Some children who could not keep still were sedated with either pentobarbital, thiopental, pentazocine, levomepromazine, risperidone, triclofos, diazepam, midazolam, or some combination of the aforementioned sedatives (Table 2). [^11^C] DPA713 was administered intravenously over 30 s by an infusion pump as scans started, and the administration dose of the trace was around 7 MBq/kg. The PET images were acquired for 60 min by Eminence SOPHIA SET-3000 BCT/X (Shimazu Co, Kyoto, Japan) in a three-dimensional acquisition mode. Before the emission scan, transmission data were acquired using a rotating Cs-137-point source for attenuation correction. The time course (0–90 min) of the asymmetry index [$\mathrm{AI}\ \left(\%\right)=200\times \frac{\mathrm{ipsilateral}\ \mathrm{VOI}\ \mathrm{value}-\mathrm{contralateral}\ \mathrm{VOI}\ \mathrm{value}}{\mathrm{ipsilateral}\ \mathrm{VOI}\ \mathrm{value}+\mathrm{contralateral}\ \mathrm{VOI}\ \mathrm{value}}$] was calculated in 14 patients with a unilateral abnormality on MRI (ipsilateral volume-of-interest [VOI]: VOI in the abnormal lesion seen on MRI, contralateral VOI: VOI in the contralateral normal area). It is found that AI was significantly elevated after administration and reached a plateau at approximately 60 min (Supplementary Figure). Therefore, the PET image frame from 40 to 60 min, which was the final stage of the elevation phase, was used for the subsequent assessment. The late plateau phase was not chosen to increase the image accuracy because we used low-dose imaging to reduce the radiation exposure and the dosage of sedative drugs for patient safety. The static PET count images were used as standardized uptake values (SUVs), which were corrected for body weight and injected activity of each patient to make SUV images. Then, the SUV images for [^11^C] DPA713PET were co-registered to the individual’s 3D T1-weighted MRI image using the Image Registration and Fusion Tool in the PMOD 3.6 software package (PMOD Technologies Ltd). The [^11^C] DPA713PET results were compared with those obtained by other neuroimaging analyses and were defined as positive or negative by a radiologist and two epileptologists.

### Measurement of [^11^C] DPA713 distribution and region of interest analysis

We made one region of interest (ROI) (10 × 10 × 10 mm^3^) for each lobe at the bilateral frontal, temporal, parietal, and occipital cortices; hippocampus; cingulate; and cerebellum and another ROI at each focal lesion in patients with definite MRI lesions using Amide’s Medical Imaging Data Examiner (AMIDE: http://amide.sourceforge.net/; Fig. 2a). After no obvious lesion was confirmed in all the cerebellums by MRI, the SUV ratio (SUVr) was calculated for an ROI/cerebellum [24]. To compare the SUVr between lesions and non-lesions, all SUVr of non-lesion ROIs and each SUVr from one lesion ROIs were averaged for each patient. Lesions were defined as (1) morphological abnormal lesion and suggesting as epileptic foci by electroencephalography (EEG) and/or MEG and (2) normal appearance lesion, observed on MRI, but suggesting as epileptic foci by EEG, MEG, and/or FDG-PET. In cases of widely affected lesions in one lobe, estimated by MRI, the ROIs in the lobe were excluded from the non-lesion ROIs.

### Immunohistochemistry

Seven patients underwent focal resection according to clinical semiology, neuroimaging, video-EEG, and electrocorticogram; the lesion was confirmed to be removed by postoperative MRI. The resected brain tissues were formalin-fixed, embedded in paraffin blocks, and sectioned at 5-μm thickness. After quenching with 0.03% hydrogen peroxide and blocking with normal serum, sections were incubated with primary antibodies (mouse monoclonal anti-human CD68 antibody [100 × dilutions; DAKO,] and mouse anti-glial fibrillary acidic protein (anti-GFAP) antibody [2000 × dilution; DAKO]) overnight, followed by appropriate biotinylated secondary antibodies (400 × dilutions) (Vector) and avidin/biotin staining (Vector ABC Elite Kit and DAB Kit, Vector Laboratories Inc). Brightfield images (magnification = 10) were obtained using a NanoZoomer 2.0RS virtual microscope (Hamamatsu photonics K.K). The CD68- or GFAP-positive area was calculated using Image J (https://imagej.net/Fiji/Downloads; threshold: 35–255 for CD68 and 200–255 for GFAP). To make Fig. 4d, e, contrast and brightness were adjusted with Photoshop software (Elements 2019, Adobe).

### Statistical analysis

The inter-rater reliability of two epileptologists was calculated by Cohen’s kappa. Average SUVs of [^11^C] DPA713PET were compared between non-lesions and lesions using the Wilcoxon rank-sum test. The correlation analysis between [^11^C] DPA713PET in non-lesions and age was performed using Spearman’s rank-order correlation analysis. The comparison of SUVr in lesions between chronic benzodiazepine (BZP) users and non-BZP users, as well as between patients with sedation and patients without sedation was performed using a two-sample *t* test. The correlation analysis between SUVr in lesions and duration of epilepsy was performed using linear regression analysis. The comparison of TSPO uptake and seizure frequency or etiology was performed using one-way analysis of variance (Bonferroni). The correlation analysis between the CD68 or GFAP-positive area and DPA713 uptake was performed using Spearman’s rank-order correlation. *P* values < 0.05 were considered significant. Statistical analysis was performed using STATA version 15.1 (Stata Corp.).

## Results

### Demographics

Twenty-seven patients (mean age: 11.3 ± 6.2 years, male/female: 17/10) were included in this study. The participants’ demographics are shown in Table 1. The mean seizure onset age was 4.2 ± 4.1 years. Twenty-two patients were diagnosed with focal epilepsy. Four patients who were diagnosed with symptomatic West syndrome showed epileptic spasms with a focal epileptogenic zone. One patient was diagnosed with Landau-Kleffner syndrome (LKS). All patients, except for one, took one or more antiepileptic drugs, including benzodiazepines (six patients). Nineteen patients had definite etiologies: tuberous sclerosis (TSC), eight; hemimegalencephaly (HME) with contralateral focal cortical dysplasia (FCD), one; FCD, one; encephalitis, two; Sturge-Weber syndrome, one; cavernous hemangioma, one; hippocampal sclerosis, one; cerebral infarction, two; low-grade glioma, one; and 1p36 deletion syndrome with pachygyria, one. Structure MRI showed focal abnormalities, such as cortical thickness, focal atrophy, and high intensity, in 18 patients. A total of 20 patients underwent interictal FDG-PET, and 15 (75.0%) patients showed focal hypometabolism. [^11^C] DPA713PET, which was performed during the interictal period, was positive in the epileptogenic zone in 23 (85.1%) patients (Table 2). All MRI-positive patients showed [^11^C] DPA713PET uptake in accordance with abnormal lesions on MRI. Five out of nine MRI-negative patients showed specific uptake patterns of [^11^C] DPA713. The inter-rater reliability between two epileptologists was 92.6% agreement (kappa = 0.63). All patients, except for one mixed binder, were high-affinity binders for TSPO SNP.

**Table 1:** Patient characteristics

Patient	Age, years	Sex	weight (kg)	Age of onset (y)	Epilepsy diagnosis (localization)	AEDs	Seizure frequency	Seizure Types	Etiology
1	17	M	53.6	6	FE (L-F)	LTG, VPA, and ZNS	Yearly	Versive seizure	–
2	10	M	35.5	5	FE (R-O)	CLB, VPA, SLM, and PER	Daily	CPS, SPS	Ulegyria
3	22	M	68.3	19	FE (R-T)	LEV	Monthly	CPS	Tumor (low grade glioma)
4	6	M	34.9	4	FE (R-F)	–	Monthly	Versive seizure	Autoimmune encephalitis
5	14	F	53.7	9	FE (R-T)	LEV and CBZ	Daily	CPS	Hippocampal sclerosis
6	4	M	15.1	0.5	WS	VPA, ZNS, ESM, and LTG	Daily	Tonic seizure, epileptic spasms	TSC
7	3	F	18.8	0.3	WS	VGB, VPA, LEV, and GBP	Daily	Tonic seizure, epileptic spasms	TSC
8	3	M	14.7	0.6	FE (L-F)	PB and LTG	Daily	hypermotor seizure	TSC2 mutation
9	13	F	29.0	0.2	FE (R-F)	LTG, VPA, AZA, LCM	Weekly	sGTC	1p36 deletion
10	9	M	24.7	5	WS	VPA and LTG	Daily	Tonic seizure, epileptic spasms	TSC
11	11	M	38.1	6	FE (L-O)	CBZ, PB, and PER	Daily/weekly	Visual SPS/sGTC	Cerebral infarction
12	16	F	47.5	3	FE (R-F)	LEV, LTG, CLB, and PER	Monthly	SPS	Cavernous hemangioma
13	25	M	48.0	0.5	FE (R-F)	TPM, CLB, LEV, and PER	Yearly	sGTC	Sturge-Weber syndrome
14	8	M	23.0	4	FE (L-F)	ZNS and CBZ	Daily	Automatism	–
15	5	M	18.6	3	FE (L-T)	PB and CZP	Daily	Epileptic spasms, CPS	Herpes encephalitis
16	11	M	35.9	7	FE (LR-F)	LTG and CBZ	Weekly	Versive seizure	–
17	16	F	43.9	4	FE (R-F)	VPA	Daily	Hypermotor seizure	TSC
18	11	M	33.7	1.6	FE (R-F)	CBZ and VPA	–	Versive seizure	TSC
19	10	M	58.5	5	LK	CZP, ESM, LCM, DZP, and VPA	Yearly	CPS	–
20	7	F	20.1	1.5	FE (LR-F)	VPA, PHT, and ZNS	Daily	Hypermotor seizure	–
21	22	F	42.0	2.3	FE (R-F)	PHT, VPA, LTG, and PER	Monthly	SPS	TSC
22	13	M	29.1	3.4	FE (L-P)	LTG, ZNS, and VPA	Daily	Hypermotor, tonic	FCD
23	19	M	61.5	13	FE (L-T)	CBZ and PER	Yearly	Visual aura~sGTC	–
24	11	M	45.6	1.3	FE (L-F)	LEV, SLM, and NZP	Weekly	brief tonic (CPS)	–
25	6	F	19.6	2.4	FE (R-F)	VPA and PER	–	subclinical seizure only	TSC
26	1	F	8.6	0.8	WS	VGB	Daily	epileptic spasms	HME+FCD
27	13	F	53.8	5.4	FE (L-F)	TPM, LEV, PER, and LTG	Weekly	SPS	–

*AEDs* antiepileptic drugs, *F* Female, *M* male, *FE* focal epilepsies, *L*- left, *R*- right, *F* frontal, *T* temporal, *O* occipital, *P* parietal, *WS* West syndrome, *LK* Landau-Kleffner syndrome, *LTG* lamotrigine, *VPA* valproic acid, *ZNS* zonisamide, *CLB* clobazam, *SLM* sultiam, *PER* perampanel, *LEV* levetiracetam, *CBZ* carbamazepine, *ESM* ethosuximide, *VGB* vigabatrin, *GBP* gabapentin, *PB* phenobarbital, *AZA* acetazolamide, *LCM* lacosamide, *TPM* topiramate, *DZP* diazepam, *PHT* phenytoin, *CPS* complex partial seizures, *SPS* simple partial seizures, *sGTC* secondarily generalized tonic–clonic convulsion, *TSC* tuberous sclerosis complex, *FCD* focal cortical dysplasias, *HME* hemimegalencephaly

**Table 2:** Neuroimaging findings and pathological data

Patients	MRI	EEG/MEG	FDG-PET^a^	[^11^C]DPA713PET increased uptake	sedation during DPA713PET	TSPO, SNP	Surgery^b^
1	Normal	L-F	Normal	No specific uptake	–	HAB	Focal ECoG (I)
2	R-O	R-TO	R-TO	R-O, R > L-T	–	HAB	Focal resection(I)
3	R-T	R-T	R-T	R-T	–	HAB	Focal resection(I)
4	atrophy	Bi-F	R-T	R-cingulate	PTB, LMP	HAB	–
5	R-T	R-T	Bi-T	R-T	–	HAB	Focal resection (I)
6	Multi (L-T)	L-CTO	N.D	L-TPO	THP,MDZ	HAB	P-quadrantectomy (III)
7	Multi	Bi-FTO	N.D	Bi-F	THP,MDZ	HAB	–
8	Normal	R-F, L-F	Normal	No specific uptake	TRF, THP	HAB	Callosotomy (IV)
9	R-FTP	R-FC	R-TP	R-FTP	MDZ,THP,PTZ	HAB	–
10	L-F, R-T	L-F	Bi-F	L-F	THP,PTZ	MAB	Focal resection (II)
11	O (R>L)	L-O	Bi-OPT	Bi-O	–	HAB	Focal resection (I→III)
12	R-F	R-C	R-FT	R-F	–	HAB	–
13	R-F	L-F	R-FT	R-F	–	HAB	–
14	Normal	Bi-F	Normal	L-F	–	HAB	–
15	L-T	Diffuse	L-T	L-T	THP	HAB	Focal resection (II)
16	Normal	L-F, R-FT	Normal	No specific uptake	THP, MDZ	HAB	–
17	MT	R-T	R-F, L-P, T	R-insula, O	DZP	HAB	Focal resection (III)
18	MT	L-T, P, O	N.D	R-F,L-F	THP,MDZ,PTZ	HAB	–
19	Normal	CSWS (R>L)	N.D	Bi-P, R-T	PTB,THP,MDZ,PTZ	HAB	- (corticosteroid effective)
20	Normal	Bi-F	L-FP	R>L insula, cingulate	PTB,THP,MDZ,PTZ	HAB	–
21	MT, SEN	R-F	N.D	R-insula~BG, L-F, P	PTB	HAB	–
22	L-TPO FCD	L-PC	L-TPO	L-TPO	RIS,PTB	HAB	–
23	Normal	L-T/L-P (MEG)	N.D	L-O, L>R hippocampus	–	HAB	EcoG L-O
24	Normal	Bi-F-Diffuse	L-hemisphere	L-FT	DZP	HAB	–
25	MT	L-F, R-F multi	N.D	R-F, L-F, cingulate	PTB	HAB	–
26	L-HME, R-TO FCD	R-TO spike	L-FTP	L-deep WM, R-OT	THP,MDZ	HAB	–
27	Normal	L-insula	Normal	No specific uptake	–	HAB	–

*MRI* magnetic resonance imaging, *EEG* electroencephalogram, *MEG* magnetoencephalography, *FDC* fludeoxyglucose F^18^, *PET* positron emission tomography, *TESP* translocator protein 18 kDa, *R* right, *SNP* single-nucleotide polymorphisms, *L* left, *P* posterior, *T* temporal, *O* occipital, *MT* multiple tubers, *HME* hemimegalencephaly, *FCD* focal cortical dysplasias, *Bi* bilateral, *BG* basal ganglia, *WM* white matter, *CSWS* continuous spikes and waves during sleep, *PTB* Pentobarbital, *THP* Thiopental, *MDZ* midazolam, *LMP* Levomepromazine, *RIS* Risperidone, *TRF* Triclofos, *PTZ* Pentazocine, *DZP* diazepam, *HAB* high-affinity binders.

### Qualitative [^11^C] DPA713PET image analysis

**Fig. 1:**
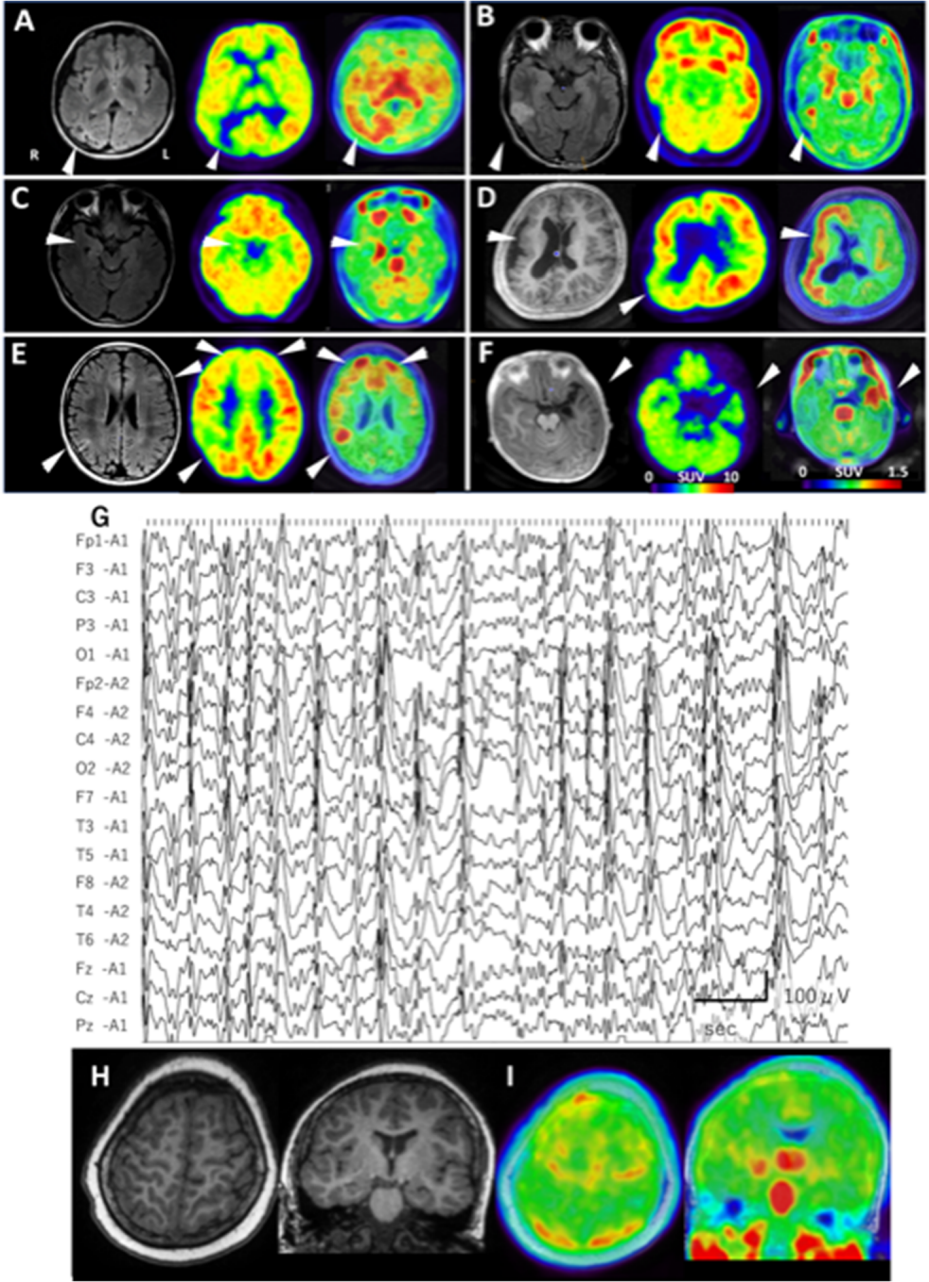
Neuroimaging data of six selected patients (**a**–**f**). Left, MRI; middle, FDG-PET; right, [^11^C] DPA713PET. **a** Patient 2. MRI showed ulegyria in the right occipital cortex. In this area, hypometabolism, as indicated by FDG-PET, and increased [^11^C] DPA713 uptake, as indicated by TSPO PET, were observed. **b** Patient 3 had low-grade gliomas with a high-intensity area in the middle temporal cortex, as indicated by FLAIR-MRI. Increased [^11^C] DPA713 uptake on TSPO PET was clearly visible compared with hypometabolism on FDG-PET. C, In patient 5, the right hippocampus showed subtle atrophy, but no obvious high intensity, on FLAIR-MRI and hypometabolism on FDG-PET. In addition, the right hippocampus showed clearly increased [^11^C] DPA713 uptake on TSPO PET. **d** Patient 9 with 1p36 deletion syndrome showed broad cortical malformation in the right hemisphere on T1-MRI. Although FDG-PET showed hypometabolism in the right temporo-occipital area and hypermetabolism in the right insula-inferior frontal area, TSPO PET demonstrated clearly increased [^11^C] DPA713 uptake in the right hemispheric cortex. **e** Patient 10 with TSC showed high intensity in the left frontal and right parietal cortex on FLAIR-MRI, suggesting cortical tubers. FDG-PET showed patchy hypometabolism, but the increased uptake area of [^11^C] DPA713 was easy to determine using TSPO PET. F, Patient 15 had atrophy caused by herpes encephalitis in the left temporal cortex and hippocampus on MRI. Although FDG-PET showed broad hypometabolism not only in the left temporal cortex but also in the right temporal cortex, TSPO PET found that the increased uptake area of [^11^C] DPA713 was restricted in the left temporal cortex. Findings obtained from patient 19, who had Landau-Kleffner syndrome (**g**–**i**). EEG showed a continuous spike-wave pattern, and the bilateral-centro-parieto-temporal discharges were predominantly observed in the right hemisphere (**g**). MRI showed no obvious abnormality (**h**), and TSPO PET revealed increased [^11^C] DPA713 uptake in the bilateral postcentral gyri and right hippocampus (**i**)

Figure 1a–f shows comparisons among MRI, FDG-PET, and [^11^C] DPA713PET images in representative six patients. Patient 2, who had ulegyria in the right occipital cortex, showed TSPO radioligand uptake in the right occipital and in the bilateral temporal area (Fig. 1a). Patient 3, who had low-grade gliomas in the right lateral temporal area, showed TSPO radioligand uptake in the tumor and in the right hippocampus (Fig. 1b). Patient 5, who had right hippocampal sclerosis, showed subtle changes on MRI and bilateral hypometabolism on FDG-PET; however, [^11^C] DPA713 uptake showed prominent increases in the right hippocampus (Fig. 1c). Patient 9, who had 1p36 deletion syndrome with right pachygyria, showed hypometabolic lesions in the right parietal and temporal cortex by FDG-PET (Fig. 1d), and [^11^C] DPA713PET clearly showed the right hemispheric distribution. In the TSC patient with multi-foci (patient 10; Fig. 1e), [^11^C] DPA713PET-positive areas were consistent with hypometabolic area indicated by FDG-PET. Although patient 15 (Fig. 1f), who had refractory seizures after herpes encephalitis, showed neuronal loss and atrophy in the left temporal area, where FDG-PET revealed broad hypometabolism, TSPO distribution was localized in the anterior temporal area.

Patient 19 had LKS, and EEG showed bilateral continuous spikes and waves predominantly in the right hemisphere (Fig. 1g). Although he showed normal MRI findings (Fig. 1h), TSPO PET found increased [^11^C] DPA713 uptake in the bilateral postcentral gyri and right hippocampus (Fig. 1i). His language cognition was improved by intravenous corticosteroid therapy.

[^11^C] DPA713PET-positive rate, which was the percentage of patients with abnormal uptake, in each etiology group were described as follows: (1) MRI-negative: 55.5% (5/9); (2) vascular lesions, such as cavernous hemangioma and Sturge-Weber syndrome: 100% (2/2); (3) gliosis (hippocampal sclerosis and post encephalopathy): 100% (3/3); (4) tumor (low-grade gliomas): 100% (1/1); and (5) cortical malformation (FCD, HME, and cortical tuber in TSC): 100% (10/10).

### Quantitative [^11^C] DPA713 binding analysis

**Fig. 2:**
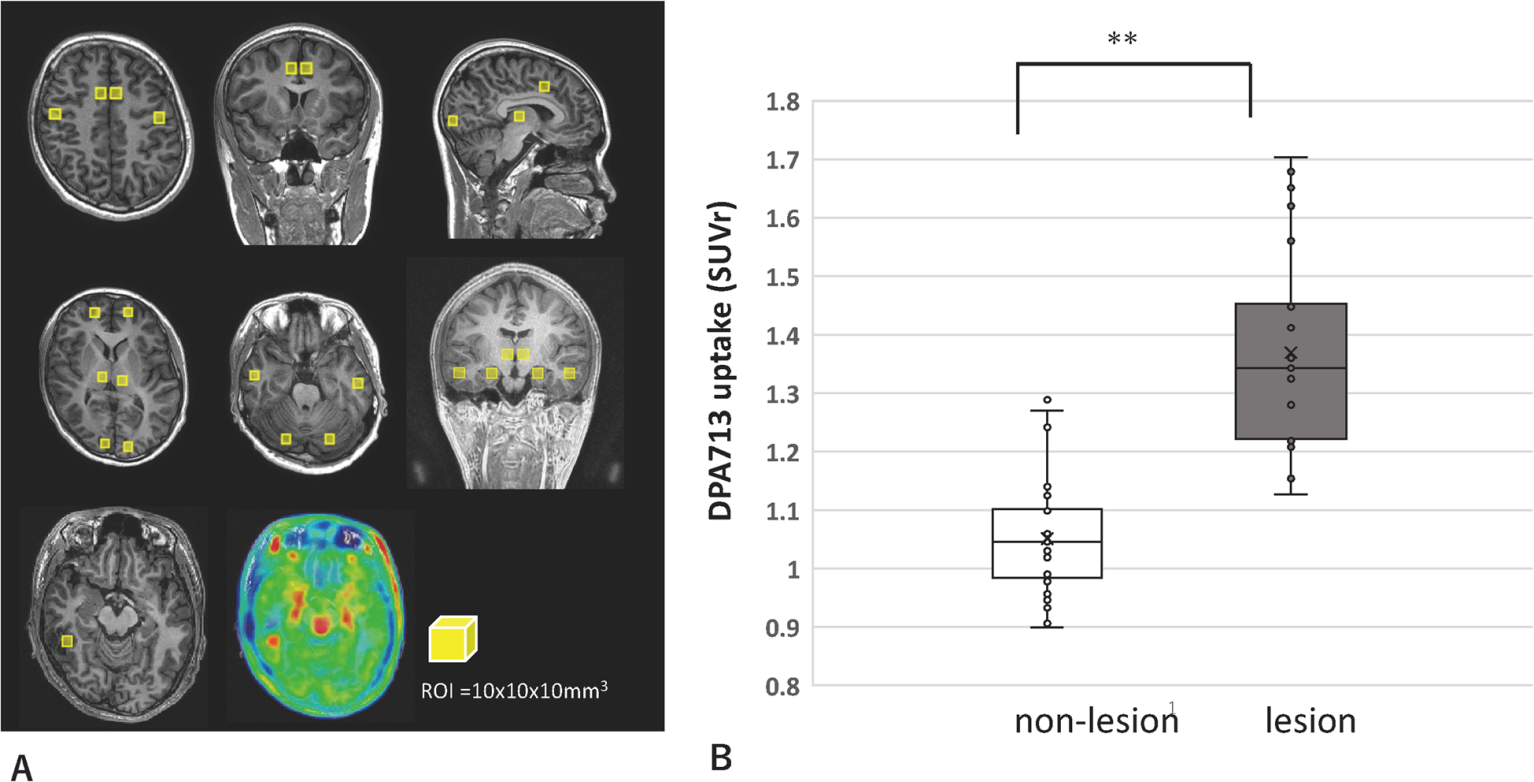
ROI-based analysis. **a** Each ROI from non-lesional cortical areas is shown in the upper and middle rows. Each ROI of DPA713PET is located on the MRI (left in bottom row) oriented merged area (middle in the bottom row). **b** Comparisons of SUVr of DPA713PET between lesions and non-lesions. The SUVr of DPA713PET was significantly higher in the lesion than in the non-lesion (*P* < 0.001)

The quantitative binding analysis revealed no significant differences in the SUVr of [^11^C] DPA713 in the non-lesion regions among different cortices (average SUVr: 0.99 ± 0.13 in the temporal cortex, 1.03 ± 0.16 in the occipital cortex, 0.96 ± 0.13 in the parietal cortex, and 0.99 ± 0.15 in the frontal cortex). In addition, the average DPA713-SUVr in non-lesions shows no association with age (*r* = − 0.366, *P* = 0.06). The average DPA713-SUVr in the lesion ROIs (1.37 ± 0.16) was significantly increased compared with that in the non-lesion ROIs (1.05 ± 0.10; Fig. 2b, *P* < 0.001).

**Fig. 3:**
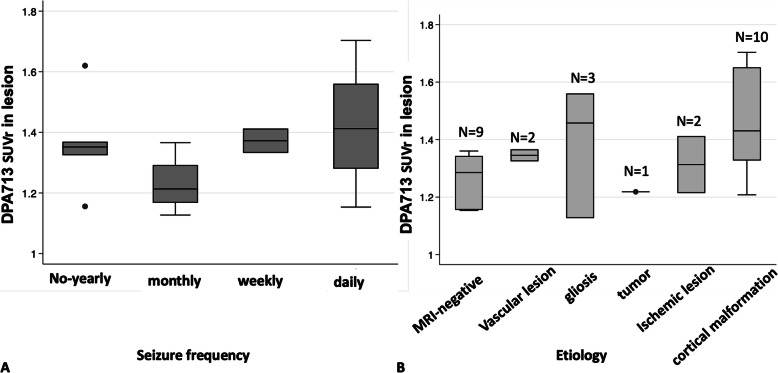
DPA713 uptake and the associated factors. DPA713 SUVr in the lesion depended on seizure frequency (**a**) and etiology (**b**)

The median DPA713-SUVr values in the lesion did not differ according to seizure frequencies (no or yearly seizure, 1.43 ± 0.19; monthly seizure, 1.44 ± 0.20; weekly seizure, 1.37 ± 0.06; daily seizure, 1.42 ± 0.19, *P* = 0.48; Fig. 3a). In addition, the median DPA713-SUVr values in the lesion did not differ between chronic BZP users and non-BZP users (1.18 ± 0.194, 1.293 ± 0.435, *P* = 0.551), and between patients with sedation and patients without sedation (1.375 ± 0.05, 1.367 ± 0.191, *P* = 0.920). Moreover, median DPA713-SUVr values in the lesion did not correlate to the duration of epilepsy (*P* = 0.669).

The DPA713-SUVr of the lesion was compared between etiologies, and it was found that DPA713-uptake tended to be most prominent in gliosis and cortical malformation, although the difference was not significant (Fig. 3b, *P* = 0.24).

**Fig. 4:**
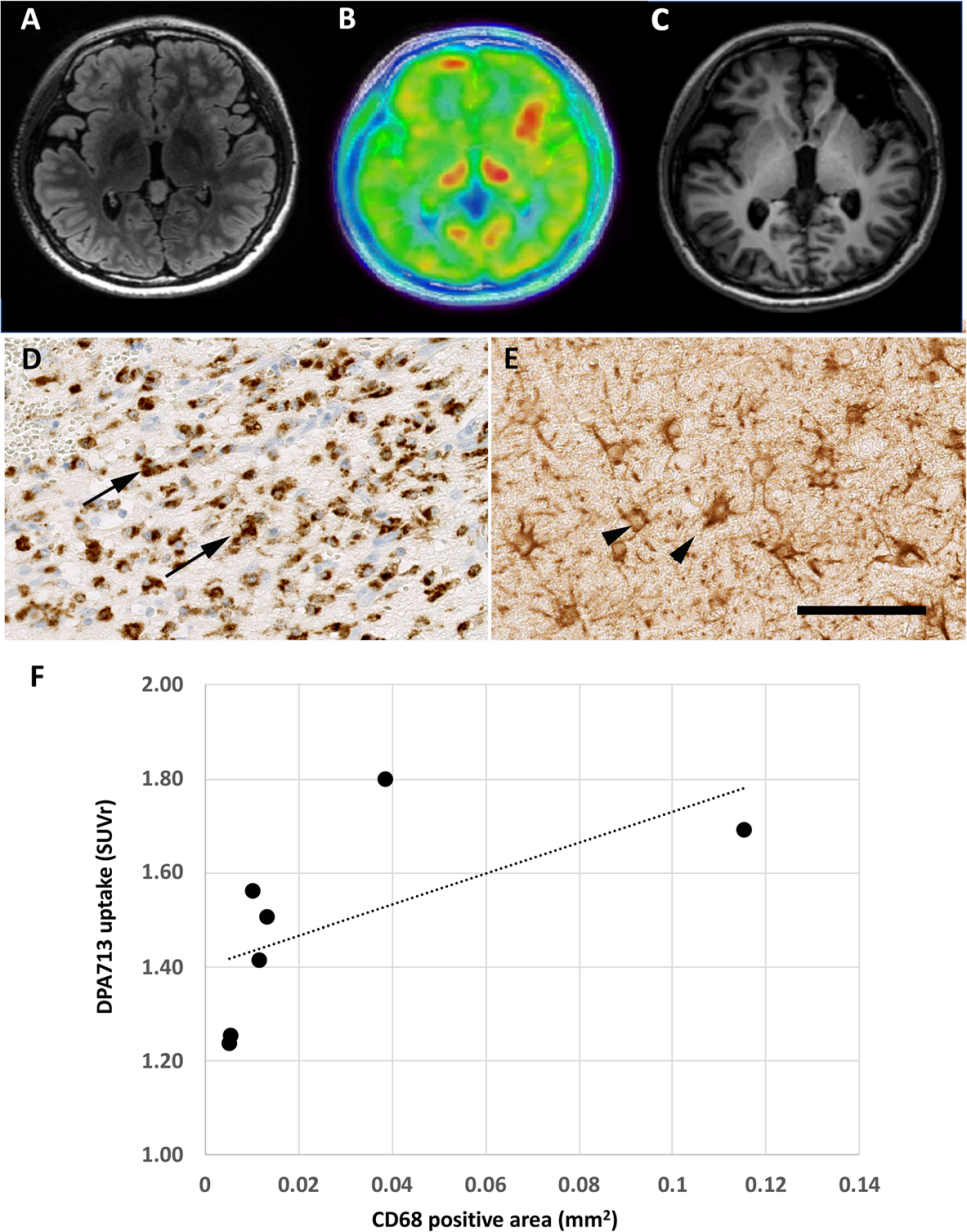
Neuroimaging and distribution of activated microglia and reactive astrocytes in the resected tissues (**a**–**b**). **a** FLAIR MRI, **b** DPA713PET, and **c** post-surgical T1-MRI for Patient 17 with TSC. This patient showed multiple cortical tubers (**a**, **b**). After examining the epileptogenic zone with subdural EEG, the focal resection was performed (**c**). In the resected tissue, immunohistochemistry for CD68 (**d**) and GFAP (**e**) showed prominent accumulation of activated microglia and reactive astrocytes. However, due to multiple lesions, although the seizure frequency was reduced, it persisted. **f** Association between the CD68 positive area and DPA713 uptake. DPA713 uptake had a positive association with the CD68 positive area (*r* = 0.857, *P* < 0.05. The *X*-axis indicates the CD68 positive area (area/mm^2^), and *Y*-axis indicates DPA uptake (SUVr). Scale bar, 100 μm

In the histopathological study, the epileptogenic lesion showed remarkable accumulation of activated microglia indicated by CD68 immunostaining and that of reactive astrocytes indicated by GFAP immunostaining in all seven patients who underwent focal resection. (Representative findings of patient 17 are presented in Fig. 4a–e.) In addition, DPA713 uptake in the lesion ROIs was significantly correlated with the CD68-positive area (*r* = 0.857, *P* < 0.05; Fig. 4f), but not with the GFAP-positive area (*r* = − 0.286, *P* = 0.53; data not shown), suggesting that increased [^11^C] DPA713 uptake correlates with the accumulation of activated microglia.

## Discussion

In this study, we found that (1) neuroinflammation defined as TSPO PET positivity was recognized in various child-onset foal epilepsies, including epileptic encephalopathy, and (2) in several resected tissues, TSPO radioligand uptake tended to be correlated with microglial accumulation. The validity of TSPO PET as a marker for neuroinflammation was confirmed by histopathological findings. To the best of our knowledge, this study is the first to demonstrate the clinical usefulness of TSPO PET in the detection of neuroinflammation in child-onset epilepsy with various etiologies.

TSPO PET has confronted problems such as high non-specific binding and the presence of low-affinity binders [25]. In this study, using [^11^C] DPA713, which is superior in the signal to noise ratio [26], we easily detected the difference in the SUVr between the non-lesion and the epileptic lesion. One previous study found that low-affinity binders accounted for 4% in the Japanese population [25]; however, there were no low-affinity binders in our present study. Therefore, TSPO PET could be very useful for neuroinflammation imaging, especially in the East Asian populations.

The brain regions with high TSPO ligand uptake in healthy controls are the pituitary gland, midbrain, thalamus, and basal ganglia, and there is relatively low TSPO ligand uptake in the cortex and cerebellum. Notably, Kumar et al. compared TSPO distribution between children and adults and found that TSPO ligand uptake in the midbrain and thalamus increased with age [23]. Specifically, TSPO PET showed an assessable signal to noise ratio in child and adolescent patients in our present study, although there was no correlation between age and TSPO ligand uptake in non-lesions (data not shown). Notably, in early child-onset epilepsy, it is difficult to determine the FCD with MRI at onset because of the immaturity of myelination [27]. Therefore, in addition to FDG-PET [28], TSPO PET could be a useful neuroimaging approach for diagnosing early-onset child epilepsy.

Neuroinflammation-associated pathological events in epilepsy include two main scenarios: (1) neuroinflammation is commonly present in the epileptogenic foci in broad etiologies, and (2) neuroinflammation is induced by repetitive epileptic activity with frequent spikes and seizure propagation in the associated brain regions. Several studies have reported the usefulness of TSPO PET in epilepsies, i.e., Rasmussen’s encephalitis [29], cerebral vasculitis [30], and intractable epilepsy due to encephalitis [31], and temporal lobe epilepsy (TLE) with hippocampal sclerosis [32]. In this study, we found that neuroinflammation contributed to focal epilepsy with wide-ranging etiologies, including tumors, ischemic lesions, and cortical malformations. Therefore, neuroinflammation in the epileptic focus might be implicated in the intractability of epilepsy.

Regarding temporal changes in microglial activation induced by seizures, [^18^F] flutriciclamide TSPO PET showed that microglial activation increased at 24 h, peaked in 5–15 days, and decreased during the chronic phase [33, 34]; moreover, it was found that subacute neuroinflammation ~ 36 h after seizures in a patient with frontal lobe epilepsy was associated with greater [^11^C]PK11195 uptake and more spatial extension in the post-seizure period than in the seizure-free period [35]. These findings suggested that microglial activation increases following a single seizure and might contribute to further epileptogenesis. In fact, microglia promote development and aggravation of epilepsy by aberrant synaptic pruning and changes in the neuronal network [36, 37]. In addition, Webster et al. have reported an age-specific vulnerability to seizures and inflammatory stimuli in children [38]. As the developing childhood brains showed higher chemokine and cytokine levels, slow glutamate clearance, and a depolarizing role of the GABA receptor, the increased seizure susceptibility may lead to high intractability in child-onset epilepsy. Our study found no significant differences in TSPO ligand uptake based on seizure frequencies, nor epilepsy duration, probably because of the different periods after last seizure, or effects of antiepileptic drugs. Although benzodiazepine drugs [39], which bind to TSPO, might affect TSPO radioligand binding, there were no differences in TSPO radioligand binding between six patients who took benzodiazepines and others who did not, and between patients with sedation and them without sedation in our study. However, as most of patients in this study showed refractory epilepsy, with obvious structural abnormality, frequent seizures, and long duration of epilepsy, the contribution of BZPs and sedative drugs to the TSPO uptake could be negligible.

Compared with FDG-PET, the increased TSPO uptake area corresponded to hypometabolic area by FDG-PET. In general, neuroinflammation increased energy consumption, where microglia upregulate GLUT1 to facilitate glucose uptake and drive the glycolysis pathway [40]. However, many previous studies reported that hypometabolism in FDG-PET is useful to localize seizure foci in various epilepsy [41] and has no correlation to neuronal loss [42]. Boison et al. reported that excessive synaptic activity causes a rapid drop in glucose, and during the excessive energy demands of seizures, astrocyte-derived lactate become an essential energy source for neurons. Therefore, patients with epilepsy are characterized by increased glucose uptake and metabolism during seizures, whereas the interictal periods are characterized by reduced glucose uptake and hypometabolism [43]. Consequently, the hypermetabolism by FDG-PET in epileptogenic zone was also reported [44].

A previous study demonstrated that TSPO ligand uptake levels were strongly correlated with the accumulation of microglia and reactive astrocytes in a rat model of TLE [45]. In our study, although the TSPO ligand uptake was positively correlated with the density of microglia, it should be noted that the sample size was small and showed large variability; TSPO ligand uptake was also not positively correlated with that of reactive astrocytes. In Brackhan’s study, the rat model was assessed at the acute phase after single status epilepticus insults [45]; however, in our study, most patients were assessed during the chronic stage. The patients whose pathology was evaluated showed frequent seizures; therefore, their TSPO PET included factors of the chronic and acute stages. In Nguyen’s study, TSPO was strongly detected in microglia and reactive astrocytes during the acute and chronic phases, respectively [46]. As Sanz [47] mentioned, epileptic seizures and inflammatory mediators in microglia and astrocytes form a vicious positive feedback loop. Further studies with larger samples are needed to verify the essential roles of microglia and astrocytes in epilepsy.

In addition, Gershen et al. reported significantly increased [^11^C] PBR28 PET uptake in the bilateral temporal regions in TLE patients compared with healthy controls [32, 48]. A study using a kainic acid-induced rat SE model found that TSPO distribution could be used to evaluate microglial activation, astrocyte reactivity, and cell loss at several time points in both acute and chronic phases. As TSPO ligand uptake increased in the epileptogenic lesion most of our patients, the usefulness of TSPO-PET for the indication of the epileptogenic zone is convincing. However, at the same time, TSPO uptake was increased in the propagated area, such as the ipsilateral hippocampus and/or contralateral hippocampus. As patients 2 and 3 were seizure free after focal resection of the right occipital lobe and the tumor in the right temporal lobe, respectively, neuroinflammation in the ipsilateral hippocampus did not suggest strong epileptogenicity. As the TSPO ligand levels increased in reactive astrocytes and microglia, TSPO PET could be used to reveal secondary gliosis along with primary inflammation [49]. Therefore, we should confirm the epileptogenicity by subdural- or stereo-EEG in the TSPO uptake area.

Temporal changes in neuroinflammation after seizures have been demonstrated [33], and TSPO PET could be used to find a good candidate for anti-inflammatory therapy. LKS is a rare form of childhood epilepsy characterized by loss of language comprehension with continuous spike and waves and rare seizures [50]. The patient with LKS in this study showed bilaterally increased uptake of TSPO ligand, corresponding with frequent EEG spikes, and the symptom was improved by the corticosteroid therapy performed after identifying neuroinflammation by TSPO PET. Further studies are needed to compare neuroinflammation between pre- and post-therapy to determine whether neuroinflammation is reduced by corticosteroids.

This study had several limitations. First, we did not obtain data regarding normal TSPO distribution in age-matched healthy children. As children are more radiosensitive than adults [51] and are at higher risk of thyroid tumors or benign pituitary adenoma [52] [53], the PET study could not be performed on healthy children, and we did not calculate the absolute radioligand value from the arterial blood samples. Second, since there are no regions in the brain that do not express TSPO [54], the reference region cannot be defined in terms of pharmacokinetics [19]. Therefore, it is impossible to calculate distribution volume or distribution volume ratios by performing pharmacokinetic analysis without arterial blood data. In both animal and clinical studies, either the thalamus [19] or cerebellum [24, 55] has been used as a pseudo-reference for calculating the SUVr. In this study, we used the cerebellum as a reference to standardize the uptake values and allow comparisons between patients. The rationale of using the cerebellum as an uptake reference is that focal epilepsy, including tuberous sclerosis, targeted in this study is known to leave no lesions in the cerebellum, and MRI did not show any abnormalities in the cerebellum in our patients. In epileptic lesions, cerebral blood flow is generally reduced during the interictal period. If the tracer uptake is biased by cerebral blood flow, it is inevitable that the uptake will decrease in epileptogenic lesions. However, DPA713 uptake increases rather than decreases in the lesions. Thus, semi-quantitative evaluation by SUVr is still useful, despite some possibility that SUVr was influenced by a severe hemispheric cortical abnormality. In this clinical study, SUVr was evaluated in the late time phase 40–60 min after the administering the ligand. When evaluating the SUV in the late static phase of [^11^C] DPA713 PET, more rigorous quantitative evaluation of brain inflammation can be performed; further studies are necessary to determine the correlation between the tracer distribution volume estimated from the full dynamic acquisition and SUV obtained from the late static phase. Moreover, we did not determine the extent of inflammation in this study. If the normal range of TSPO uptake is known, it will help in assessing the extent of the inflammation. Third, the sample size was not enough to compare TSPO ligand uptake among different etiologies, and the number of patients whose pathology could be examined was also limited. Therefore, further studies are needed to confirm our present findings in patients undergoing surgical intervention and those with focal epilepsy with a variety of etiologies.

## Conclusions

This study found that [^11^C] DPA713 PET showed valuable sensitivity for detecting focal epileptogenic zones in child-onset focal epilepsy of various etiologies. Although the increased uptake of [^11^C] DPA713, which was significantly associated with microglial accumulation, revealed that neuroinflammation is a common neuropathological feature in child-onset focal epilepsy, the causal link to its refractory nature and the influence on cognitive functions remain unclear. Further research is needed to evaluate [^11^C] DPA713 in longitudinal studies and whole-brain analyses.

## Supplementary Information

**Additional file 1: Supplementary Figure**. A) Sphere volume-of-interest (VOI) (φ5-15mm) was located on the epileptogenic foci resected by surgery or on the pathological region determined by MRI and on the corresponding region in the contralateral hemisphere. If there were multiple pathological lesions (e.g. cortical tuber), the lesion included in the epileptogenic zone was selected as the focus, and the contralateral control VOI was located in the normal apparent area based on the co-registered MRI. B) The time course of the AI of the patients were averaged. The graph shows the two groups comprising 14 subjects with unilateral foci: 10 patients were imaged from 0 to 60 minutes, and the remaining 4 patients were scanned from 30 to 90 minutes after the administration of the ligand. Bars represent the standard error of the mean

## Data Availability

The datasets used and analyzed during the current study are available from the corresponding author on reasonable request.

## Abbreviations

EEG: Electroencephalography
PET: Positron emission tomography
MRI: Magnetic resonance imaging
SUV: Standard uptake value
SUVr: Standardized uptake value ratio
TSPO: Translocator protein
DPA: *N*,*N*-diethyl-2-(4-methoxyphenyl)-5,7-dimethylpyrazolo[1,5-a]pyrimidine-3-acetamide
FDG-PET: ^18^F-2-deoxy-2-fluoro-d-glucose positron emission tomography
VOI: Volume-of-interest
ROI: Region of interest
CD68: Cluster of differentiation 68
GFAP: Glial fibrillary acidic protein
LKS: Landau-Kleffner syndrome
TSC: Tuberous sclerosis
HME: Hemimegalencephaly
FCD: Focal cortical dysplasia
TLE: Temporal lobe epilepsy
