# Supplementary figures and images for: Clinical evaluation of neuroinflammation in child-onset focal epilepsy: a translocator protein PET study
Source: J Neuroinflammation. 2021 Jan 6;18:8. doi: 10.1186/s12974-020-02055-1 (PMC7789379; doi:10.1186/s12974-020-02055-1)

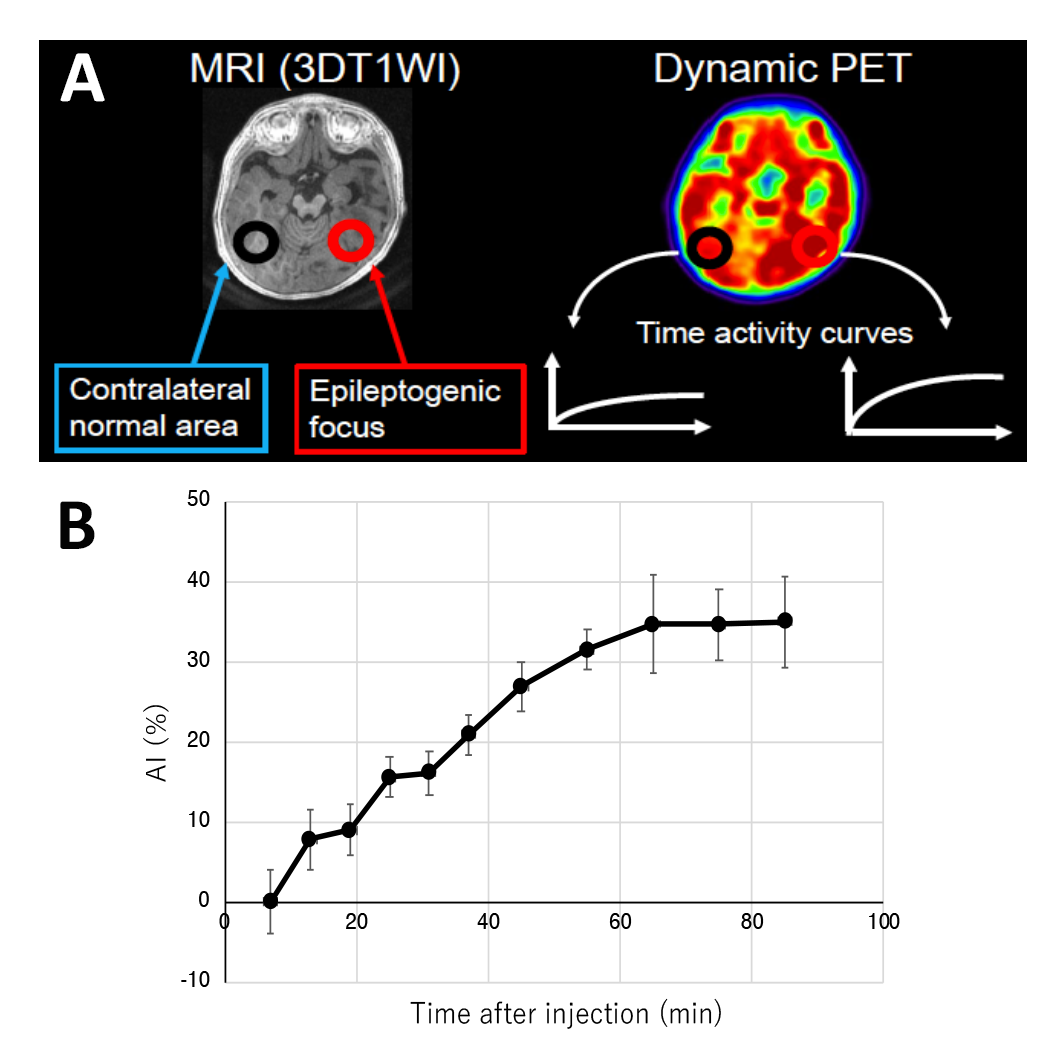

Supplement: Supplementary file 1 — Additional file 1: Supplementary Figure. A) Sphere volume-of-interest (VOI) (φ5-15mm) was located on the epileptogenic foci resected by surgery or on the pathological region determined by MRI and on the corresponding region in the contralateral hemisphere. If there were multiple pathological lesions (e.g. cortical tuber), the lesion included in the epileptogenic zone was selected as the focus, and the contralateral control VOI was located in the normal apparent area based on the co-registered MRI. B) The time course of the AI of the patients were averaged. The graph shows the two groups comprising 14 subjects with unilateral foci: 10 patients were imaged from 0 to 60 minutes, and the remaining 4 patients were scanned from 30 to 90 minutes after the administration of the ligand. Bars represent the standard error of the mean [file 12974_2020_2055_MOESM1_ESM.tif]
